# Domain structure of ultrathin ferromagnetic elements in the presence of Dzyaloshinskii–Moriya interaction

**DOI:** 10.1098/rspa.2016.0666

**Published:** 2017-01

**Authors:** Cyrill B. Muratov, Valeriy V. Slastikov

**Affiliations:** 1 Department of Mathematical Sciences, New Jersey Institute of Technology, Newark, NJ 07102, USA; 2 School of Mathematics, University of Bristol, Bristol BS8 1TW, UK

**Keywords:** micromagnetics, Dzyaloshinskii–Moria interaction, chiral domain walls, magnetic skyrmions, gradient theory of phase transitions, *Γ*-convergence

## Abstract

Recent advances in nanofabrication make it possible to produce multilayer nanostructures composed of ultrathin film materials with thickness down to a few monolayers of atoms and lateral extent of several tens of nanometers. At these scales, ferromagnetic materials begin to exhibit unusual properties, such as perpendicular magnetocrystalline anisotropy and antisymmetric exchange, also referred to as Dzyaloshinskii–Moriya interaction (DMI), because of the increased importance of interfacial effects. The presence of surface DMI has been demonstrated to fundamentally alter the structure of domain walls. Here we use the micromagnetic modelling framework to analyse the existence and structure of chiral domain walls, viewed as minimizers of a suitable micromagnetic energy functional. We explicitly construct the minimizers in the one-dimensional setting, both for the interior and edge walls, for a broad range of parameters. We then use the methods of *Γ*-convergence to analyse the asymptotics of the two-dimensional magnetization patterns in samples of large spatial extent in the presence of weak applied magnetic fields.

## 1. Introduction

The exploding amount of today’s digital data calls for revolutionary new high-density, fast and long-term information storage solutions. Spintronics is one among the emerging fields of nanotechnology offering a great promise for information technologies, whereby information is carried and processed, using the electron spin rather than its electric charge [1–4]. It brings about many opportunities for creating the next generation of devices combining spin-dependent effects with conventional charge-based electronics. Despite being a relatively new field of applied physics, it has already firmly established its presence in everyday life through the development of new magnetic storage devices. The discovery of giant magnetoresistance (GMR), for which A. Fert and P. Grünberg were awarded the 2007 Nobel Prize in Physics, allowed an ability to ‘read’ the magnetization states of a ferromagnet through electric resistance measurements. This effect has been used in GMR-based spin valves, which transformed magnetic hard-disk drive technology, leading to increases in storage density by several orders of magnitude. Yet, the GMR magnetic storage technology has already been superseded by novel spin-dependent devices based on the effect of tunnelling magnetoresistance, another exciting development in the field of spintronics [4].

Recent discoveries of new physical phenomena that become prominent at the nanoscale open up a possibility of unprecedented data storage densities and read/write speeds. These include spin transfer torque (STT), chiral domain walls and magnetic skyrmions, spin Hall effect, spin Seebeck effect, electric field control of the magnetic properties, etc. (e.g. [4–10]). The ability to manipulate the magnetization using electric currents suggests novel designs for magnetic memory. One popular concept is the so-called racetrack memory [4,11], which uses a two-dimensional array of parallel nanowires where magnetic domain ‘bits’ may be read, moved and written through an application of a spin current. Another promising type of memory and logic device is based on storing and manipulating the data bits, using magnetic skyrmions, rather than magnetic domain walls. The existence of magnetic skyrmions was predicted theoretically more than 25 years ago [12,13], but their experimental observations are much more recent [7,14,15]. The topological stability, small size and extremely low currents and fields required to move magnetic skyrmions make them natural candidates for the use in spintronic memory and logic devices [6,15,16].

A successful design of novel spintronic devices that make use of magnetic domain walls or skyrmions is strongly dependent on a deep theoretical understanding of static and dynamic behaviours of the magnetization in magnetic nanostructures. The manipulation and control of magnetic domain walls and topologically protected states (e.g. magnetic vortices and skyrmions) in ferromagnetic nanostructures has been the subject of extensive experimental and theoretical research (e.g. [8,17–22]; this list is certainly far from complete). Recent advances in nanofabrication techniques [23] have led to the production of ultrathin films with thickness down to several atomic layers and a lateral extent down to tens of nanometers. These ultrathin magnetic films and multilayer structures often exhibit unusual magnetic properties, attributed to an increased importance of interfacial effects. The most important features of these ultrathin magnetic structures include the appearance of perpendicular magnetic anisotropy [24,25] and the Dzyaloshinskii–Moriya interaction (DMI) [26,27]. The latter is closely related to reflection symmetry breaking in such films and leads to emergence of magnetization chirality [18,28,29].

**Figure 1.**
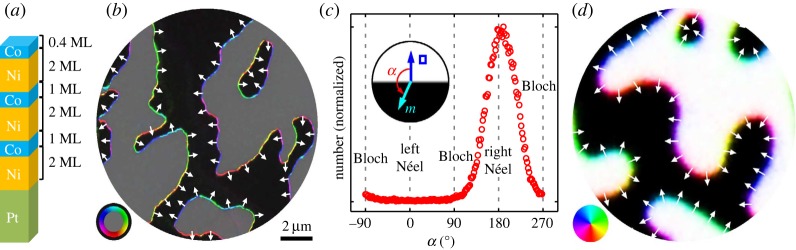
Experimental and numerical observations of chiral domain walls in ultrathin ferromagnetic films in the presence of DMI. (*a*) The schematics of the multilayer structure (ML, monolayer). (*b*) A colourmap of the magnetization exhibiting chiral domain walls. (*c*) A histogram of the in-plane magnetization orientation angle relative to the in-plane normal to the domain wall showing a preferred rotation direction. (*d*) A comparison to the result of a Monte Carlo simulation of a discrete spin model. In (*b*), grey indicates the domains with the magnetization up, black indicates the domains with the magnetization down and the rest of the colours correspond to the directions of the in-plane component, as shown in the colour-wheel. Adapted from [8], with permission; see that reference for further details. (Online version in colour.)

The experimental discovery of the symmetry breaking DMI in ferromagnetic multilayers has generated a lot of interest in the physics community [14,30,31]. There has been a lot of work focusing on the influence of DMI on magnetization configurations within a ferromagnetic sample [18,19,30]. One of the interesting features of DMI is its influence on the profile and the dynamic properties of domain walls [8,18,19,32]. In addition, it is well-known that DMI may be responsible for formation of magnetic skyrmions—topologically protected states with a quantized topological degree observed in ultrathin films [7,33]. DMI also plays a crucial role in defining the orientation of the domain walls and chiral behaviour of the magnetization inside the wall, leading to the formation of a new type of *chiral domain walls*, also referred to as Dzyaloshinskii walls [18], having rather different properties than the conventional Bloch and Neel walls [34]. For an illustration of chiral domain walls observed experimentally and numerically, see figure 1. In a recent theoretical work [19], it was reported that the interplay between DMI and the boundary of an ultrathin ferromagnetic sample is responsible for creating another type of domain wall—*chiral edge domain walls*. These walls play a crucial role in producing new types of magnetization patterns inside a ferromagnet. For instance, in the presence of a transverse applied field, chiral edge domain walls provide a mechanism for *tilting* of an interior domain wall in a ferromagnetic strip [22,35]. Moreover, they also significantly modify the dynamic behaviour of the interior domain wall under the action of current and an applied field [18].

In this paper, we study chiral domain walls in ultrathin ferromagnetic films, using rigorous analytical methods within the variational framework of micromagnetics. Our goal is to understand the formation of chiral interior domain walls and chiral edge domain walls, viewed as local or global energy minimizing configurations of the magnetization, in samples with perpendicular magnetocrystalline anisotropy in the presence of surface DMI and weak applied magnetic fields. The multi-scale nature of the micromagnetic energy allows for a variety of distinct regimes characterized by different relations between the material and geometric parameters, and makes its investigation a very challenging mathematical problem. Many of these regimes have been investigated analytically, using modern techniques of calculus of variations in the context of various ferromagnetics nanostructures (e.g. [36]).

Our starting point is a reduced two-dimensional micromagnetic energy, in which the stray field contributes only a local shape anisotropy term to the leading order (see (2.2)). This energy gives rise to a non-convex vectorial variational problem, with a non-trivial interplay between the boundary and the interior of the domain due to the DMI term. We seek to understand the formation and structure of the domain walls—transition layers between constant magnetization states—that correspond to minimizers of the micromagnetic energy. The framework for this analysis is provided by the variational methods of the gradient theory of phase transitions [37]. These types of problems have been extensively studied in the mathematical community in both scalar [37–40] and vectorial [41,42] settings. The non-trivial influence of the boundary within the gradient theory of phase transitions was investigated in [38,40].

We begin by investigating the one-dimensional problems on the infinite and semi-infinite domains. Here we provide a complete analytical solution for the global energy minimizers of these one-dimensional problems, see theorems 3.1 and 3.4, respectively. Our main tool is a careful analysis of the case of equality in the vectorial Modica–Mortola type lower bound for the energy of one-dimensional magnetization configurations. Our analysis yields explicit profiles for one-dimensional chiral interior and edge domain walls. These optimal profiles are used later on in the constructions for the full two-dimensional problem. Our one-dimensional results confirm the physical intuition of [19] for a slightly reduced range of the DMI constants.

We then investigate the full two-dimensional energy in the regime of large domains and small applied fields, using methods of *Γ*-convergence. After a rescaling, this amounts to a study of the asymptotic behaviour of the energy *E*_*ε*_(**m**) in (4.2) as *ε*→0. We note that our original problem is vectorial, constrained (|**m**(*x*)|=1), and the energy contains linear gradient terms in the interior, as well as boundary terms (after integration by parts), both coming from DMI. Even though the original problem is vectorial—and these are notoriously difficult phase transition problems—we show that one can reduce our problem to a scalar setting by decoupling the behaviour of the normal magnetization component *m*_∥_, preferring to be equal to ±1, and the in-plane component **m**_⊥_, preferring to be 0, outside the transition layer and proving that the optimal configuration of **m**_⊥_ is a function of *m*_∥_ and the layer orientation. This non-trivial observation significantly simplifies the analysis of the problem and allows us to use the methods developed in [38,40] to obtain the *Γ*-limit of the family of micromagnetic energies. The rest of the proof follows the pattern of the gradient theory of phase transitions [37], with some modifications to account for the vectorial and constrained nature of the problem.

With the above tools, we obtain the *Γ*-limit, given by (4.3), of the family of energies in (4.2) with respect to the *L*^1^ convergence of $m_{∥}^{\varepsilon }$. The limit energy is geometric, and its minimizers determine the locations of the chiral domain walls, which are now curves separating the regions in which $m_{∥}^{0}$ changes sign. As a consequence, we also obtain an asymptotic characterization of the energy minimizers of *E*_*ε*_ as *ε*→0. Our main result, stated in theorem 4.1, indicates that the presence of DMI significantly modifies the magnetization behaviour in ultrathin magnetic films by creating both interior and edge chiral domain walls.

The paper is organized as follows. In §2, we introduce the basic micromagnetic modelling framework. In §3, we present the solution of the one-dimensional global energy minimization problem for both the interior and boundary chiral domain walls. Then, in §4, we investigate the full two-dimensional energy (2.2) in the regime of large domains and small applied fields and study the behaviour of the family of micromagnetic energies in (4.2) in the limit as *ε*→0. Finally, in §5, we summarize our findings and discuss several additional modelling aspects of our problem, together with some possible extensions of our analysis.

## 2. Model

We start by considering a ferromagnetic film of thickness *d* occupying the spatial domain $\Omega \times (0,d)\subset R^{3}$, where $\Omega \subseteq R^{2}$ is a two-dimensional domain specifying the shape of the ferromagnetic element. Within the micromagnetic framework [34], the magnetization in the sample is described by the vector **M**=**M**(*x*,*y*,*z*) of constant length |**M**|=*M*_*s*_, where *M*_*s*_ is referred to as the saturation magnetization. The micromagnetic energy in the presence of an out-of-plane uniaxial anisotropy and an interfacial DMI may be written in the SI units in the form [12,13,18]

2.1 $$\begin{array}{rl} E(\text{M}) & =\frac{A}{M_{s}^{2}}\int_{\Omega \times (0,d)}|\nabla \text{M}{|}^{2}\;d^{3}r+\frac{K}{M_{s}^{2}}\int_{\Omega \times (0,d)}|{\text{M}}_{⊥}{|}^{2}\;d^{3}r-\mu_{0}\int_{\Omega \times (0,d)}\text{M}\cdot \text{H}\;d^{3}r\\ & \;+\mu_{0}\int_{R^{3}}\int_{R^{3}}\frac{\nabla \cdot \text{M}(\text{r})\nabla \cdot \text{M}({\text{r}}^{\prime })}{8\pi |\text{r}-{\text{r}}^{\prime }|}\;d^{3}r\;d^{3}r^{\prime }+\frac{Dd}{M_{s}^{2}}\int_{\Omega }({\bar{M}}_{∥}\nabla \cdot {\bar{\text{M}}}_{⊥}-{\bar{\text{M}}}_{⊥}\cdot \nabla {\bar{M}}_{∥})\;d^{2}r. \end{array}$$

Here we wrote **M**=(**M**_⊥_,*M*_∥_), where we defined ${\text{M}}_{⊥}\in R^{2}$ and $M_{∥}\in R$ to be the components of the magnetization vector **M** that are perpendicular and parallel to the material easy axis (the *z*-axis), respectively, and introduced $\bar{\text{M}}$ which is the trace of **M** on *Ω*×{0}. In (2.1), *A* is the exchange stiffness, *K* is the magnetocrystalline anisotropy constant, **M** has been extended by zero outside the sample and ∇⋅**M** is understood distributionally in $R^{3}$, *μ*_0_ is the permeability of vacuum, **H**=**H**(*x*,*y*,*z*) is the applied magnetic field and *D* is the DMI constant, following the standard convention to write *D* in the units of energy per unit area. In writing the DMI term in this specific form, we took into account that it arises as a contribution from the interface between the magnetic layer and a non-magnetic material and should, therefore, enter as a boundary term in the full three-dimensional theory.

In the above framework, the equilibrium magnetization configurations in the ferromagnetic sample correspond to either global or local minimizers of a non-local, non-convex energy functional in (2.1). This energy includes several terms, in order of appearance: the exchange term, which prefers constant magnetization configurations; the magnetocrystalline anisotropy, which favours out-of-plane magnetization configurations; the Zeeman, or applied field term, which prefers magnetizations aligned with the external field; the magnetostatic term, which prefers divergence-free configurations; and the surface DMI term, which favours chiral symmetry breaking. The origin of the latter is the antisymmetric exchange mediated by the spin-orbit coupling in the conduction band of a heavy metal at the ferromagnet–metal interface [43,28,44].

The variational problem associated with (2.1) poses a significant challenge for analysis. Therefore, in the following, we introduce a simplified version of the energy in (2.1) that is suitable for ultrathin ferromagnetic films of thickness $d≲\ell_{\mathrm{ex}}=\sqrt{2A/(\mu_{0}M_{s}^{2})}$, where ℓ_*ex*_ is the material exchange length. In this case, a two-dimensional model is appropriate in which the stray field energy can be modelled by a local shape anisotropy term (e.g. [45]; for a more thorough mathematical discussion of the stray field effect in ultrathin films with perpendicular anisotropy, see [46]). Measuring the lengths in the units of ℓ_*ex*_ and the energy in the units of *Ad*, we can rewrite the energy associated with the magnetization configuration **M**(*x*,*y*,*z*)=*M*_*s*_**m**(*x*,*y*), where $\text{m}:\Omega \to S^{2}$, as

2.2 $$\begin{array}{r} E(\text{m})=\int_{\Omega }\{|\nabla \text{m}{|}^{2}+(Q-1)|{\text{m}}_{⊥}{|}^{2}-2h_{∥}m_{∥}-2{\text{h}}_{⊥}\cdot {\text{m}}_{⊥}+\kappa (m_{∥}\nabla \cdot {\text{m}}_{⊥}-{\text{m}}_{⊥}\cdot \nabla m_{∥})\}\;d^{2}r, \end{array}$$

where we defined ${\text{m}}_{⊥}\in R^{2}$ and $m_{∥}\in R$ to be the respective components of the unit magnetization vector **m** and introduced the dimensionless quality factor *Q* and the dimensionless DMI strength *κ*:

2.3 $$Q=\frac{2K}{\mu_{0}M_{s}^{2}},\;\kappa =D\sqrt{\frac{2}{\mu_{0}M_{s}^{2}A}},$$

where *D* is the DMI constant [18]. In (2.2), we also introduced a dimensionless applied magnetic field **h**=(**h**_⊥_,*h*_∥_)=**H**/*M*_*s*_, with ${\text{h}}_{⊥}\in R^{2}$ and $h_{∥}\in R$.

We are interested in the regime in which the film favours magnetizations that are normal to the film plane, i.e. when *Q*>1. Also, as the energy is invariant with respect to the transformation

2.4 $$\kappa \to -\kappa ,\;{\text{m}}_{⊥}\to -{\text{m}}_{⊥}\;\mathrm{and}\;{\text{h}}_{⊥}\to -{\text{h}}_{⊥},$$

without loss of generality, we can assume *κ* to be positive.

## 3. The problem in one dimension

**Figure 2.**
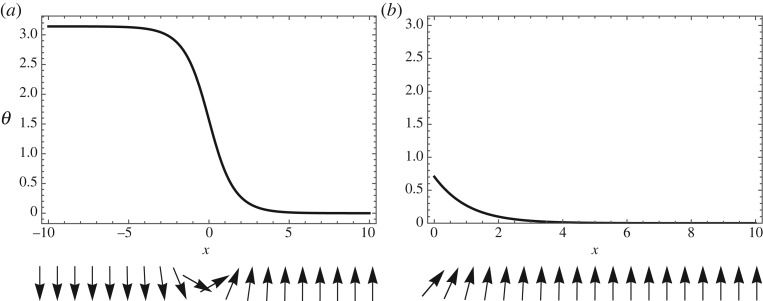
Two types of one-dimensional domain walls due to DMI: (*a*) interior wall and (*b*) edge wall. In the upper panels, *θ* stands for the angle between **m** and the *z*-axis. The vector **m** rotates in the *xz*-plane (lower panels).

We begin by considering an idealized situation in which the ferromagnetic film occupies either the whole plane or a half-plane, which leads to two basic types of domain walls considered below (figure 2). These are the magnetization configurations that vary in one direction only. In the case of the half-plane, the magnetization is also assumed to vary in the direction normal to the film edge. Throughout this section, we set the applied magnetic field **h** to zero.

### (a) Interior wall

Consider first the whole space situation, in which case we may assume that

3.1 $$\Omega =\{(x,y)\in R^{2}\;:\;x\in R,\;0<y<1\},$$

with periodic boundary conditions at *y*=0 and *y*=1. We then take **m** to be a one-dimensional profile, i.e. **m**=**m**(*x*). Then we may write the energy associated with **m** in the form

3.2 $$E(\text{m})=\int_{-\infty }^{\infty }\{|{\text{m}}^{\prime }{|}^{2}+(Q-1)|{\text{m}}_{⊥}{|}^{2}+\kappa (m_{∥}{\left(\hat{x}\cdot {\text{m}}_{⊥}\right)}^{\prime }-(\hat{x}\cdot {\text{m}}_{⊥})m_{∥}^{\prime })\}\;dx,$$

where primes denote the derivative with respect to the *x* variable and $\hat{x}$ is the unit vector in the direction of the *x*-axis. We are interested in the global energy minimizers of the energy in (3.2) that obey the following conditions at infinity:

3.3 $$\lim_{x\to \pm \infty }m_{∥}(x)=\pm 1\;\mathrm{and}\;\lim_{x\to \pm \infty }{\text{m}}_{⊥}(x)=0.$$

On heuristic grounds, one expects that the optimal domain wall profile has the form of the *Dzyaloshinskii wall* [18]. Namely, one expects that in the domain wall the magnetization rotates around the direction of the *y*-axis. Hence, introducing an ansatz

3.4 m=(sin⁡θ,0,cos⁡θ),

one can rewrite the energy in (3.2) as [19]

3.5 $$E(\text{m})=\int_{-\infty }^{\infty }\{|\theta^{\prime }{|}^{2}+(Q-1)\sin^{2}\theta +\kappa \theta^{\prime }\}\;dx.$$

Observe, however, that *a priori* the energy in (3.5) is not well defined in the natural class of $\theta \in H_{\mathrm{loc}}^{1}(R)$, as the last term in the energy is not sign definite and does not necessarily make sense as the Lebesgue integral on the whole real line. This fact is closely related to the chiral nature of DMI, favouring oscillations of the magnetization vector. A simple counterexample, in which the first two terms of the energy in (3.5) are well defined, while the last one is not, is given by the function *θ*(*x*)=*π*/2−*Si*(*x*), where $\mathrm{Si}(x)=\int_{0}^{x}t^{-1}\sin t\;dt$ is the sine integral function. It is also worth noting that if one were to define the energy in (3.5) as the limit of the energies on large finite domains, then its minimum value would be strictly less than that obtained from the integral on the whole real line due to the presence of edge domain walls [19] (see also §3b for further details).

To fix the issue above, one needs to assume that $\theta^{\prime }\in L^{1}(R)$, which introduces a bound on the total variation of *θ* on R. This, in turn, implies that the limit of *θ*(*x*) as x→±∞ exists, and the last term in (3.5) becomes a boundary term. Furthermore, in order for the energy to be bounded the limits of *θ*(*x*) at infinity must be integer multiples of *π*, and without loss of generality we may assume

3.6 $$\lim_{x\to -\infty }\theta (x)=\pi n\;\mathrm{and}\;\lim_{x\to +\infty }\theta (x)=0,\;n\in Z.$$

The energy then becomes

3.7 $$E(\text{m})=\int_{-\infty }^{\infty }\{|\theta^{\prime }{|}^{2}+(Q-1)\sin^{2}\theta \}\;dx-\kappa \pi n,$$

for $\theta \in H_{\mathrm{loc}}^{1}(R)$ with $\theta^{\prime }\in L^{1}(R)$ and *θ* obeying (3.6).

It is easy to see that the energy in (3.7) is uniquely minimized in the above class if and only if *n*=1 and *κ*<*κ*_*c*_, where

3.8 $$\kappa_{c}=\frac{4\sqrt{Q-1}}{\pi }.$$

In this case, the optimal profile is, up to translations, given by [19]

3.9 $$\theta (x)=2\arctan\;e^{-x\sqrt{Q-1}},$$

and the wall energy is given by

3.10 $$\sigma_{\mathrm{wall}}=4\sqrt{Q-1}-\pi \kappa >0.$$

Indeed, minimizers of (3.7) with *n*=±1 among all admissible *θ* are well known to exist due to the good coercivity and lower semicontinuity properties of those terms (for technical details in a related problem, see [47]). The profile in (3.9) is then the unique solution, up to translations and sign, of the Euler–Lagrange equation associated with (3.7) satisfying (3.6). At the same time, for |*n*|≥2 the energy is easily seen to satisfy *E*(*θ*)≥|*n*|*σ*_*wall*_. Hence, by inspection the minimizer with *n*=+1 corresponds to the global minimizer for all *n*≠0, with the sign of *n* corresponding to the wall chirality imparted by DMI.

We remark that, in contrast with the above situation, the problem associated with (3.2) does not admit minimizers for *κ*>*κ*_*c*_, as in this case the energy is not bounded below and favours helical structures [19].

The following theorem establishes existence and uniqueness of the minimizers of the one-dimensional domain wall energy in (3.2) among all profiles satisfying (3.3) *without* assuming the ansatz in (3.4). In view of the discussion above, an appropriate admissible class for the energy is given by

3.11 $$A=\{\text{m}\in H_{\mathrm{loc}}^{1}(R;S^{2})\;:\;{\text{m}}^{\prime }\in L^{1}(R;R^{3})\}.$$

The theorem below confirms the expectation that the domain wall profile is given by (3.4) and (3.9) for all *κ* below a critical value, although the latter turns out to be slightly lower than the expected threshold value of *κ*=*κ*_*c*_ given by (3.8).

**Theorem 3.1.**

*Let* $0<\kappa <\sqrt{Q-1}$. *Then there exists a unique, up to translations, minimizer* m∈A *of* (*3.2*) *satisfying* (*3.3*). *The minimizer* m *has the form in* (*3.4*) *with θ given by* (*3.9*), *and the minimal energy is given by σ*_*wall*_ *from* (*3.10*).

**Proof.**

The proof proceeds by showing directly that the profile given by (3.4) and (3.9) is the unique minimizer via establishing a sharp lower bound for the energy. Assume without loss of generality that E(m)<+∞. Then by dominated convergence theorem, we have

3.12 $$\begin{array}{r} E(\text{m})=\int_{-\infty }^{\infty }(|{\text{m}}^{\prime }{|}^{2}+(Q-1)|{\text{m}}_{⊥}{|}^{2})\;dx+\kappa \lim_{R\to \infty }\int_{-R}^{R}(m_{∥}{\left(\hat{x}\cdot {\text{m}}_{⊥}\right)}^{\prime }-(\hat{x}\cdot {\text{m}}_{⊥})m_{∥}^{\prime })\;dx, \end{array}$$

and |**m**_⊥_(*x*)|→0 as x→±∞ [48], Corollary 8.9. Using integration by parts [48], Corollary 8.10, the last integral may be rewritten as

3.13 $$\int_{-R}^{R}(m_{∥}{\left(\hat{x}\cdot {\text{m}}_{⊥}\right)}^{\prime }-(\hat{x}\cdot {\text{m}}_{⊥})m_{∥}^{\prime })\;dx=(\hat{x}\cdot {\text{m}}_{⊥}(x))m_{∥}(x){|}_{-R}^{R}-2\int_{-R}^{R}(\hat{x}\cdot {\text{m}}_{⊥})m_{∥}^{\prime }\;dx.$$

Therefore, passing to the limit we obtain that

3.14 $$E(\text{m})=\int_{-\infty }^{\infty }(|{\text{m}}^{\prime }{|}^{2}+(Q-1)|{\text{m}}_{⊥}{|}^{2}-2\kappa (\hat{x}\cdot {\text{m}}_{⊥})m_{∥}^{\prime })\;dx.$$

We now trivially estimate the DMI term from below to obtain

3.15 $$E(\text{m})\geq \int_{-\infty }^{\infty }(|{\text{m}}^{\prime }{|}^{2}+(Q-1)|{\text{m}}_{⊥}{|}^{2}-2\kappa |{\text{m}}_{⊥}|\;|m_{∥}^{\prime }|)\;dx.$$

Next, we use the standard trick [49] to estimate the exchange energy by the term involving only |*m*_∥_′|. In the following, we spell out the details of the argument, paying special attention to the optimality of the obtained estimates. We start by applying the weak chain rule [48], Proposition 9.5 to the identity $|{\text{m}}_{⊥}{|}^{2}+m_{∥}^{2}=1$. This yields

3.16 $$m_{∥}^{2}|m_{∥}^{\prime }{|}^{2}=|{\text{m}}_{⊥}\cdot {\text{m}}_{⊥}^{\prime }{|}^{2}\leq |{\text{m}}_{⊥}{|}^{2}|{\text{m}}_{⊥}^{\prime }{|}^{2}\;\text{for a.e.}\;x\in R.$$

Therefore, for a.e. x∈R such that |*m*_∥_|<1, we can write

3.17 $$\frac{m_{∥}^{2}|m_{∥}^{\prime }{|}^{2}}{1-m_{∥}^{2}}\leq |{\text{m}}_{⊥}^{\prime }{|}^{2}.$$

Thus

3.18 $$\int_{-\infty }^{\infty }|{\text{m}}^{\prime }{|}^{2}\;dx=\int_{-\infty }^{\infty }(|{\text{m}}_{⊥}^{\prime }{|}^{2}+|m_{∥}^{\prime }{|}^{2})\;dx\geq \int_{\left\{|m_{∥}|<1\right\}}\frac{|m_{∥}^{\prime }{|}^{2}}{1-m_{∥}^{2}}\;dx.$$

Writing the lower bound for the energy in terms of *m*_∥_, with the help of (3.15) and (3.18) we obtain

3.19 $$E(\text{m})\geq \int_{\left\{|m_{∥}|<1\right\}}\left(\frac{|m_{∥}^{\prime }{|}^{2}}{1-m_{∥}^{2}}+(Q-1)(1-m_{∥}^{2})\right)\;dx-2\kappa \int_{-\infty }^{\infty }\sqrt{1-m_{∥}^{2}}|m_{∥}^{\prime }|\;dx.$$

This inequality may be rewritten in the following Modica–Mortola type form

3.20 $$\begin{array}{rl} E(\text{m}) & \geq 2\int_{-\infty }^{\infty }(\sqrt{Q-1}-\kappa \sqrt{1-m_{∥}^{2}})|m_{∥}^{\prime }|\;dx\\ & \;+\int_{\left\{|m_{∥}|<1\right\}}{\left(\frac{|m_{∥}^{\prime }|}{\sqrt{1-m_{∥}^{2}}}-\sqrt{\left(Q-1)(1-m_{∥}^{2}\right)}\right)}^{2}\;dx, \end{array}$$

where we extended the domain of integration in the first term to the whole real line in view of the fact that by (3.16) we have *m*_∥_′=0 whenever |*m*_∥_|=1.

We now turn to showing that the energy is minimized by the profile given by (3.4) with *θ* given by (3.9). Indeed, from (3.20) we have for any *R*>0

3.21 $$\begin{array}{rl} E(\text{m}) & \geq 2\int_{-R}^{R}(\sqrt{Q-1}-\kappa \sqrt{1-m_{∥}^{2}})|m_{∥}^{\prime }|\;dx\\ & \geq 2\int_{-R}^{R}(\sqrt{Q-1}-\kappa \sqrt{1-m_{∥}^{2}})m_{∥}^{\prime }\;dx\\ & =\{2m_{∥}(x)\sqrt{Q-1}-\kappa (m_{∥}(x)\sqrt{1-m_{∥}^{2}(x)}+\arcsin(m_{∥}(x)))\}{|}_{-R}^{R}, \end{array}$$

where we used the assumption that $\kappa <\sqrt{Q-1}$ to go from the first to the second line. Finally, passing to the limit as R→∞ and using (3.3), we obtain

3.22 $$E(\text{m})\geq \sigma_{\mathrm{wall}},$$

where *σ*_*wall*_ is defined in (3.10). At the same time, by the computation at the beginning of this section the inequality above is an equality when **m** is given by (3.4) with *θ* from (3.9).

It remains to prove that the profile given by (3.4) with *θ* from (3.9) is the unique, up to translations, minimizer of the energy that satisfies (3.3). Without loss of generality, we may assume that *m*_∥_(0)=0, in view of the continuity of *m*_∥_(*x*) and (3.3). As the minimal value of the energy is attained by dropping the last term in (3.20) and replacing |*m*_∥_′| with *m*_∥_′, we have *m*_∥_′(*x*)≥0 for a.e. x∈R, and *m*_∥_ satisfies

3.23 $$m_{∥}^{\prime }=\sqrt{Q-1}(1-m_{∥}^{2})\;\text{for a.e.}\;x\in I,$$

where *I*=(*a*,*b*) with −∞≤a<0<b≤∞. As the right-hand side of (3.23) is continuous, *m*_∥_ is the unique classical solution of (3.23) that satisfies *m*_∥_(0)=0, which is explicitly $m_{∥}(x)=\tanh(x\sqrt{Q-1})$. Lastly, the inequality in (3.16) becomes equality when **m**_⊥_′ is parallel to **m**_⊥_ and, hence, **m**_⊥_=*g***b** for some constant vector $\text{b}\in R^{2}$ and a scalar function g:R→[−1,1]. In turn, to make an inequality in (3.15) an equality, one needs to choose $\text{b}=\hat{x}$ and *g*≥0. In view of the unit length constraint for |**m**|, this translates into ${\text{m}}_{⊥}=\hat{x}\;{\mathrm{sech}}^{2}(x\sqrt{Q-1})$. The obtained profile **m**=(**m**_⊥_,*m*_∥_) is then precisely the one given by (3.4) with *θ* from (3.9). ▪

We note that the arguments in the proof of theorem 3.1 do not carry over to the range $\sqrt{Q-1}<\kappa \leq \kappa_{c}$, as in this range we can no longer reduce the energy by passing to the configurations in the form given by (3.4). Nevertheless, an inspection of the proof shows that the statement of theorem 3.1 remains true for all **m**=(**m**_⊥_,*m*_∥_) such that *m*_∥_(*x*) is a non-decreasing function of *x*. Hence, we have the following result.

**Theorem 3.2**

*For any κ*>0, *there exists a unique, up to translations, minimizer of* (*3.2*) *among all* $m=(m_{⊥},m_{∥})\in A$ *satisfying* (*3.3*) *and m*_∥_′≥0. *The minimizer* m *has the form in* (*3.4*) *with θ given by* (*3.9*), *and the minimal energy is given by σ*_wall_ *from* (*3.10*).

**Remark 3.3**

We point out that due to the presence of the edge domain walls (see the following subsection) the minimizers of the energy in (2.2) in the form of a Dzyaloshinskii wall on a strip Ω=R×(0,L) are not one dimensional for any *L*>0. Nevertheless, if one assumes periodic boundary conditions instead of the natural boundary conditions at the edges of the strip, an examination of the proof of theorem 3.1 shows that the global minimizer is still given by (3.4) and (3.9) in this case.

### (b) Edge wall

Consider now the half-plane situation, in which case we may assume that

3.24 $$\Omega =\{(x,y)\in R^{2}\;:\;x>0,\;0<y<1\},$$

with periodic boundary conditions at *y*=0 and *y*=1. Taking **m** to be a one-dimensional profile, i.e. **m**=**m**(*x*), we write

3.25 $$E(\text{m})=\int_{0}^{\infty }\{|{\text{m}}^{\prime }{|}^{2}+(Q-1)|{\text{m}}_{⊥}{|}^{2}+\kappa (m_{∥}{\left(\hat{x}\cdot {\text{m}}_{⊥}\right)}^{\prime }-(\hat{x}\cdot {\text{m}}_{⊥})m_{∥}^{\prime })\}\;dx,$$

where, as before, $\hat{x}$ is the unit vector in the direction of the *x*-axis. Once again, in order for this energy to be bounded, we must have |**m**_⊥_(*x*)|→0 as x→∞. Hence, in view of the symmetry

3.26 $${\text{m}}_{⊥}\to -{\text{m}}_{⊥}\;\mathrm{and}\;m_{∥}\to -m_{∥},$$

without loss of generality we may assume that

3.27 $$\lim_{x\to \infty }m_{∥}(x)=1.$$

Note, however, that the value of **m**(0) is not fixed and needs to be determined for the optimal domain wall profile at the material edge. Such edge domains walls were first discussed in [19] (for closely related objects in bulk helimagnets, see also [50,51]).

As for *κ*>*κ*_*c*_, where *κ*_*c*_ is given by (3.8), the energy favours helical structures [19] and, hence, is not bounded below on the semi-infinite interval as well as on the whole line, throughout the rest of this section we assume that *κ*<*κ*_*c*_. Assuming also the ansatz from (3.4) and arguing as in the previous subsection, for $\theta \in H^{1}(R^{+})$ with $\theta^{\prime }\in L^{1}(R^{+})$ we may write the energy in (3.25) as

3.28 $$E(\text{m})=\int_{0}^{\infty }\{|\theta^{\prime }{|}^{2}+(Q-1)\sin^{2}\theta \}\;dx-\kappa \theta (0),$$

which is easily seen to be minimized at fixed *θ*(0)=*θ*_0_∈(0,*π*) by

3.29 $$\theta (x)=2\arctan\;e^{(x_{0}-x)\sqrt{Q-1}},\;x_{0}=\frac{\ln\tan(\theta_{0}/2)}{\sqrt{Q-1}}.$$

Indeed, using the Modica–Mortola trick [37], we rewrite the energy in (3.28) as

3.30 $$\begin{array}{rl} E(\text{m}) & =2\sqrt{Q-1}\int_{0}^{\infty }|\sin\theta |\;|\theta^{\prime }|\;dx+\int_{0}^{\infty }{\left(|\theta^{\prime }|-\sqrt{Q-1}|\sin\theta |\right)}^{2}\;dx-\kappa \theta_{0}\\ & \geq -\int_{0}^{\infty }(2\sqrt{Q-1}|\sin\theta |-\kappa )\theta^{\prime }\;dx=\int_{0}^{\theta_{0}}(2\sqrt{Q-1}|\sin\theta |-\kappa )\;d\theta . \end{array}$$

In particular, the inequality above becomes an equality when *θ* is given by (3.29).

We now show that there exists a unique value of $\theta_{0}=\theta_{0}^{*}\in (0,\pi )$ for which the function from (3.29) yields the absolute minimum of the energy in (3.28) for *κ*<*κ*_c_. Denoting the right-hand side in (3.30) by *F*(*θ*_0_), we observe that *F*(0)=0, *F*′(0)<0 and *F*(*θ*_0_)=*F*(*θ*_0_−*π*)+*σ*_wall_, where *σ*_wall_>0 is given by (3.10), for all *θ*_0_≥*π*. Therefore, for *θ*_0_≥0 it is enough to consider the values of *θ*_0_∈(0,*π*), for which we have explicitly

3.31 $$F(\theta_{0})=2\sqrt{Q-1}(1-\cos\theta_{0})-\kappa \theta_{0}.$$

A simple computation then shows that for *θ*_0_≥0 the function *F*(*θ*_0_) is uniquely minimized by

3.32 $$\theta_{0}^{*}=\arcsin\left(\frac{\kappa }{2\sqrt{Q-1}}\right),$$

and the minimal value of *F*(*θ*_0_) is given by

3.33 $$\sigma_{\mathrm{edge}}=2\sqrt{Q-1}\left(1-\sqrt{1-\frac{\kappa^{2}}{4(Q-1)}}\right)-\kappa \arcsin\left(\frac{\kappa }{2\sqrt{Q-1}}\right)<0.$$

In fact, this is also an absolute lower bound for *E*(**m**) in (3.28), as for *θ*_0_<0 the energy remains positive. Furthermore, as $\theta_{0}^{*}\in (0,\pi )$, this minimum value is attained by the profile in (3.29) with $\theta_{0}=\theta_{0}^{*}$. Interestingly, we find that $\theta_{0}^{*}\in (0,\arcsin(2/\pi ))$, spanning the range from 0^°^ at *κ*=0 to about 39.5^°^ for *κ*=*κ*_c_. Thus, the global minimizer of the energy in (3.25) among all profiles satisfying (3.4) has the form of an edge domain wall whose profile is given by (3.29), up to a sign, with an optimal value of *θ* at the edge.

We now prove, once again, that this picture remains true without the ansatz in (3.4) for a slightly smaller range of the values of *κ*<*κ*_c_. The appropriate admissible class for the energy in (3.25) is now

3.34 $$A^{+}=\{\text{m}\in H_{\mathrm{loc}}^{1}(R^{+};S^{2})\;:\;{\text{m}}^{\prime }\in L^{1}(R^{+};R^{3})\}.$$

**Theorem 3.4.**

*Let* $0<\kappa <\sqrt{Q-1}$. *Then there exists a unique minimizer* $m\in A^{+}$ *of* (*3.25*) *satisfying* (*3.27*). *The minimizer* m *has the form in* (*3.4*) *with θ given by* (*3.29*) *and* $\theta_{0}=\theta_{0}^{*}$ *from* (*3.32*), *and the minimal energy is given by σ*_edge_ *from* (*3.33*).

**Proof.**

The proof proceeds exactly as in the case of theorem 3.1, except that there is now an extra contribution from the boundary of the domain at *x*=0. Namely, instead of (3.14) we obtain

3.35 $$E(\text{m})=\int_{0}^{\infty }(|{\text{m}}^{\prime }{|}^{2}+(Q-1)|{\text{m}}_{⊥}{|}^{2}-2\kappa (\hat{x}\cdot {\text{m}}_{⊥})m_{∥}^{\prime })\;dx-\kappa m_{∥}(0)(\hat{x}\cdot {\text{m}}_{⊥}(0)).$$

Estimating both terms coming from DMI from below as

3.36 $$E(\text{m})\geq \int_{0}^{\infty }(|{\text{m}}^{\prime }{|}^{2}+(Q-1)|{\text{m}}_{⊥}{|}^{2}-2\kappa |{\text{m}}_{⊥}|\;|m_{∥}^{\prime }|)\;dx-\kappa |m_{∥}(0)|\;|{\text{m}}_{⊥}(0)|,$$

and retracing the steps in the proof of theorem 3.1, we obtain

3.37 $$\begin{array}{rl} E(\text{m}) & \geq 2\int_{0}^{\infty }(\sqrt{Q-1}-\kappa \sqrt{1-m_{∥}^{2}})|m_{∥}^{\prime }|\;dx-\kappa |m_{∥}(0)|\sqrt{1-m_{∥}^{2}(0)}\\ & \;+\int_{\left\{|m_{∥}|<1\right\}}{\left(\frac{|m_{∥}^{\prime }|}{\sqrt{1-m_{∥}^{2}}}-\sqrt{\left(Q-1)(1-m_{∥}^{2}\right)}\right)}^{2}\;dx. \end{array}$$

With the help of the identity |*m*_∥_′|=| |*m*_∥_|′|[52], Theorem 6.17 and our assumption on *κ*, we can further estimate the right-hand side in (3.37) from below as

3.38 $$\begin{array}{rl} E(\text{m}) & \geq 2\int_{0}^{R}(\sqrt{Q-1}-\kappa \sqrt{1-m_{∥}^{2}})|m_{∥}^{\prime }|\;dx-\kappa |m_{∥}(0)|\sqrt{1-m_{∥}^{2}(0)}\\ & \geq 2\int_{0}^{R}(\sqrt{Q-1}-\kappa \sqrt{1-m_{∥}^{2}})|m_{∥}{|}^{\prime }\;dx-\kappa |m_{∥}(0)|\sqrt{1-m_{∥}^{2}(0)}\\ & =\{2|m_{∥}(x)|\sqrt{Q-1}-\kappa (|m_{∥}(x)|\sqrt{1-m_{∥}^{2}(x)}+\arcsin(|m_{∥}(x)|))\}{|}_{0}^{R}\\ & \;-\kappa |m_{∥}(0)|\sqrt{1-m_{∥}^{2}(0)}. \end{array}$$

Simplifying the expression above and passing to the limit, we arrive at

3.39 $$E(\text{m})\geq 2\sqrt{Q-1}(1-|m_{∥}(0)|)-\kappa \arccos|m_{∥}(0)|.$$

However, the right-hand side of (3.39) is nothing but $F(\arccos|m_{∥}(0)|)$, where *F* is given by (3.31). Thus, *E*(**m**)≥*σ*_edge_, and equality holds for the profile given by (3.4) and (3.29). Furthermore, as in the case of theorem 3.1, the inequality above is strict for any other wall profile. This concludes the proof. ▪

**Remark 3.5**

According to theorem 3.4, the magnetization vector in the edge wall that asymptotes to *m*_∥_=+1 in the sample interior acquires a component that points along the inner normal at the sample edge. At the same time, by (3.26) the magnetization vector in the edge wall that asymptotes to *m*_∥_=−1 in the sample interior acquires a component that points along the outer normal at the sample edge.

## 4. The problem in two dimensions

**Figure 3.**
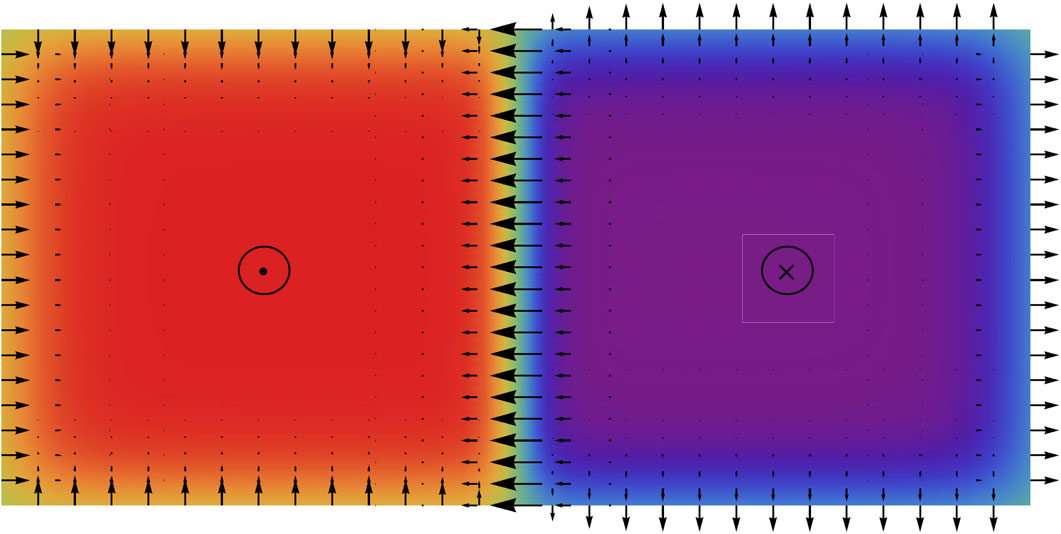
Schematics of a magnetization configuration containing edge walls and a Dzyaloshinskii wall. The arrows show the in-plane components of the magnetization vector, the colours correspond to the out-of-plane component (‘red’ is up, ‘violet’ is down, also indicated by up/down symbols). (Online version in colour.)

We now go back to the original two-dimensional problem and consider the regime in which the Dzyaloshinskii domain walls are present (for an illustration, see figure 3). The appearance of these domain walls requires that the lateral extent of the ferromagnetic sample be sufficiently large. Therefore, we introduce the domain *Ω*_*ε*_=*ε*^−1^*Ω*, where *ε*≪1, and redefine the energy in (2.2) on *Ω*_*ε*_:

4.1 $$E(\text{m})=\int_{\Omega_{\varepsilon }}\{|\nabla \text{m}{|}^{2}+(Q-1)|{\text{m}}_{⊥}{|}^{2}-2{\text{h}}_{\varepsilon }\cdot \text{m}+\kappa (m_{∥}\nabla \cdot {\text{m}}_{⊥}-{\text{m}}_{⊥}\cdot \nabla m_{∥})\}\;d^{2}r,$$

where we also defined a rescaled applied field ${\text{h}}_{\varepsilon }=({\text{h}}_{⊥}^{\varepsilon },h_{∥}^{\varepsilon })=\varepsilon ({\text{h}}_{⊥}^{0},h_{∥}^{0})=\varepsilon {\text{h}}_{0}$, chosen to have an appropriate balance between the Zeeman and the domain wall energies (see below). We then rescale the domain back to *Ω* and the energy by a factor of *ε*, which leads to the following family of energies:

4.2 $$\begin{array}{rl} E_{\varepsilon }(\text{m}) & =\int_{\Omega }\{\varepsilon |\nabla \text{m}{|}^{2}+\varepsilon^{-1}(Q-1)|{\text{m}}_{⊥}{|}^{2}-2h_{∥}^{0}m_{∥}-2{\text{h}}_{⊥}^{0}\cdot {\text{m}}_{⊥}\\ & \;+\kappa (m_{∥}\nabla \cdot {\text{m}}_{⊥}-{\text{m}}_{⊥}\cdot \nabla m_{∥})\}\;d^{2}r. \end{array}$$

The purpose of this section is to understand the behaviour of global energy minimizers of *E*_*ε*_ as *ε*→0, which corresponds to the regime of interest. Throughout the rest of this paper, $\Omega \subset R^{2}$ is assumed to be a bounded domain with boundary of class *C*^2^. This is done merely to reduce the technicalities of the proofs and focus on the vectorial aspects of the problem involving DMI. With slight modifications, the proof should apply to the case when ∂*Ω* is a union of finitely many curve segments of class *C*^1^ (see also [38], Remark 1.3).

Our main tool for the analysis of the variational problem associated with (4.2) will be the following *Γ*-convergence result.

**Theorem 4.1.**

*Let* $h_{0}=(h_{⊥}^{0},h_{∥}^{0})\in L^{\infty }(\Omega ;R^{3}),$ *Q*>1 *and* $0<\kappa <\sqrt{Q-1}$. *Then, as ε*→0, *we have E*_*ε*_→*ΓE*_0_ *with respect to the L*^1^ *convergence, where*

4.3 $$E_{0}(m_{∥})=\sigma_{\mathrm{edge}}H^{1}(\partial \Omega )+\sigma_{\mathrm{wall}}H^{1}(\partial^{*}\Omega^{+})-2\int_{\Omega }h_{∥}^{0}m_{∥}\;d^{2}r,$$

*in which m*_∥_∈*BV* (*Ω*;{−1,1}) *and* ∂*Ω^+^ *is the reduced boundary of the set Ω*^+^, *where*

4.4 $$\Omega^{\pm }=\{x\in \Omega \;:\;m_{∥}(x)=\pm 1\}.$$

*More precisely:*

(*i*) *For any sequence of* $m_{\varepsilon }=(m_{⊥}^{\varepsilon },m_{∥}^{\varepsilon })\in H^{1}(\Omega ;S^{2})$ *such that* $\underset{\varepsilon \to 0}{lim sup}E_{\varepsilon }(m_{\varepsilon })<+\infty$ *there is a subsequence (not relabelled) and a function* $m_{∥}^{0}\in BV(\Omega ;\{-1,1\})$ *such that* $m_{∥}^{\varepsilon }\to m_{∥}^{0}$ *and* $|m_{⊥}^{\varepsilon }|\to 0$ *in L*^1^*(Ω) as ε→0, and
*
  4.5 $$\underset{\varepsilon \to 0}{lim inf}E_{\varepsilon }(m_{\varepsilon })\geq E_{0}(m_{∥}^{0}).$$
(*ii*) *For any* $m_{∥}^{0}\in BV(\Omega ;\{-1,1\})$ *there is a sequence of* $m_{\varepsilon }=(m_{⊥}^{\varepsilon },m_{∥}^{\varepsilon })\in H^{1}(\Omega ;S^{2})$ *such that* $m_{∥}^{\varepsilon }\to m_{∥}^{0}$ *and* $|m_{⊥}^{\varepsilon }|\to 0$ *in L*^1^*(Ω) as ε→0, and
*
  4.6 $$\underset{\varepsilon \to 0}{lim sup}E_{\varepsilon }(m_{\varepsilon })\leq E_{0}(m_{∥}^{0}).$$

**Proof.**

The proof follows the classical argument of Modica [38] adapted to the vectorial micromagnetic setting and taking into account the boundary contributions to the energy. The latter arise after integration by parts:

4.7 $$\begin{array}{rl} E_{\varepsilon }(\text{m}) & =\int_{\Omega }(\varepsilon |\nabla \text{m}{|}^{2}+\varepsilon^{-1}(Q-1)|{\text{m}}_{⊥}{|}^{2}-2h_{∥}^{0}m_{∥}-2{\text{h}}_{⊥}^{0}\cdot {\text{m}}_{⊥}^{0}-2\kappa {\text{m}}_{⊥}\cdot \nabla m_{∥})\;d^{2}r\\ & \;+\kappa \int_{\partial \Omega }{\tilde{m}}_{∥}({\tilde{\text{m}}}_{⊥}\cdot \nu )\;dH^{1}(r), \end{array}$$

where *ν* is the outward unit normal to ∂*Ω* and $\left({\tilde{\text{m}}}_{⊥},{\tilde{m}}_{∥}\right)$ is the trace of (**m**_⊥_,*m*_∥_) on ∂*Ω*. The proof proceeds in three steps.

*Step 1*: *Compactness*. Given an admissible sequence of ${\text{m}}_{\varepsilon }=({\text{m}}_{⊥}^{\varepsilon },m_{∥}^{\varepsilon })$ satisfying *E*_*ε*_(**m**_*ε*_)≤*C* as *ε*→0 for some *C*>0 independent of *ε*, with the help of (4.7) and an elementary bound on the DMI term we can write

4.8 $$\begin{array}{r} \int_{\Omega }(\varepsilon |\nabla {\text{m}}_{\varepsilon }{|}^{2}+\varepsilon^{-1}(Q-1)|{\text{m}}_{⊥}^{\varepsilon }{|}^{2}-2\kappa |{\text{m}}_{⊥}^{\varepsilon }|\;|\nabla m_{∥}^{\varepsilon }|)\;d^{2}r\leq C+2∥|{\text{h}}_{0}|{∥}_{L^{\infty }(\Omega )}|\Omega |+\kappa H^{1}(\partial \Omega ). \end{array}$$

Therefore, from (3.17) we obtain

4.9 $$\begin{array}{rl} & \int_{\Omega \cap \{|m_{∥}^{\varepsilon }|<1\}}\left(\frac{\varepsilon |\nabla m_{∥}^{\varepsilon }{|}^{2}}{1-|m_{∥}^{\varepsilon }{|}^{2}}+\varepsilon^{-1}(Q-1)(1-|m_{∥}^{\varepsilon }{|}^{2})\right)\;d^{2}r\\ & \;-2\kappa \int_{\Omega }\sqrt{1-|m_{∥}^{\varepsilon }{|}^{2}}\;|\nabla m_{∥}^{\varepsilon }|\;d^{2}r\leq C^{\prime }, \end{array}$$

for some constant *C*′>0 independent of *ε*. Applying the Modica–Mortola trick to the first line in (4.9) and using the fact that by (3.16) we have $|\nabla m_{∥}^{\varepsilon }|=0$ whenever $|m_{∥}^{\varepsilon }|=1$, we obtain

4.10 $$\begin{array}{r} 2\int_{\Omega }(\sqrt{Q-1}-\kappa \sqrt{1-|m_{∥}^{\varepsilon }{|}^{2}})|\nabla m_{∥}^{\varepsilon }|\;d^{2}r\leq C^{\prime }. \end{array}$$

This is equivalent to $\int_{\Omega }|\nabla \Phi (m_{∥}^{\varepsilon })|\;d^{2}r\leq C^{\prime }$, where

4.11 $$\Phi (s)=2\int_{0}^{s}(\sqrt{Q-1}-\kappa \sqrt{1-t^{2}})\;dt=2s\sqrt{Q-1}-\kappa s\sqrt{1-s^{2}}-\kappa \arcsin s$$

is a continuously differentiable, strictly increasing odd function of *s*∈[−1,1]. Furthermore, by our assumption on *κ* we have $0<2(\sqrt{Q-1}-\kappa )\leq \Phi^{\prime }(s)\leq 2\sqrt{Q-1}$. Therefore, by weak chain rule [48], Proposition 9.5 we have

4.12 $$\begin{array}{r} ∥m_{∥}^{\varepsilon }{∥}_{W^{1,1}(\Omega )}\leq C^{\prime \prime }, \end{array}$$

for some *C*′′>0 independent of *ε*. In turn, by compactness in *BV* (*Ω*) and the compact embedding of *BV* (*Ω*) into *L*^1^(*Ω*) [53], this yields, upon extraction of a subsequence, that $m_{∥}^{\varepsilon }\to m_{∥}^{0}$ in *L*^1^(*Ω*) for some $m_{∥}^{0}\in BV(\Omega )$.

To prove that $|m_{∥}^{0}|=1$ and, as a consequence, that $|{\text{m}}_{⊥}^{\varepsilon }|\to 0$ in *L*^1^(*Ω*), we combine (4.9) and (4.12) to get

4.13 $$\begin{array}{r} \varepsilon^{-1}(Q-1)\int_{\Omega }(1-|m_{∥}^{\varepsilon }{|}^{2})\;d^{2}r\leq C^{\prime }+2\kappa C^{\prime \prime }. \end{array}$$

Therefore, the integral in the left-hand side of (4.13) converges to zero as *ε*→0 and, hence, $m_{∥}^{\varepsilon }(x)\to \pm 1$ for a.e. *x*∈*Ω*. This concludes the proof of the compactness part of our *Γ*-convergence result.

*Step 2*: *Lower bound*. We now proceed to establish (4.5). By the Modica–Mortola type arguments in Step 1, we can estimate the energy from below as

4.14 $$E_{\varepsilon }({\text{m}}_{\varepsilon })\geq \int_{\Omega }(|\nabla \Phi (m_{∥}^{\varepsilon })|-2h_{∥}^{0}m_{∥}^{\varepsilon }-2{\text{h}}_{⊥}^{0}\cdot {\text{m}}_{⊥}^{\varepsilon })\;d^{2}r-\kappa \int_{\partial \Omega }|{\tilde{m}}_{∥}^{\varepsilon }|\sqrt{1-|{\tilde{m}}_{∥}^{\varepsilon }{|}^{2}}\;dH^{1}(r).$$

Let $u_{\varepsilon }=\Phi (m_{∥}^{\varepsilon })$. Then the lower bound in (4.14) may be rewritten as

4.15 $$E_{\varepsilon }({\text{m}}_{\varepsilon })\geq \int_{\Omega }(|\nabla u_{\varepsilon }|-2h_{∥}^{0}m_{∥}^{\varepsilon }-2{\text{h}}_{⊥}^{0}\cdot {\text{m}}_{⊥}^{\varepsilon })\;d^{2}r+\int_{\partial \Omega }\sigma ({\tilde{u}}_{\varepsilon })\;dH^{1}(r),$$

where $\sigma (u)=-\kappa |\Phi^{-1}(u)|\sqrt{1-|\Phi^{-1}(u){|}^{2}}$ and ${\tilde{u}}_{\varepsilon }$ is the trace of *u*_*ε*_ on ∂*Ω*, noting that *u*=*Φ*(*s*) defines a continuously differentiable one-to-one map from [−1,1] to $I=[-2\sqrt{Q-1}+\frac{1}{2}\pi \kappa ,2\sqrt{Q-1}-\frac{1}{2}\pi \kappa ]$. We next define

4.16 $$\tilde{\sigma }(u)=|u|+\min_{t\in I}(\sigma (t)-|t|)\;u\in I.$$

A straightforward calculation shows that we have explicitly

4.17 $$\tilde{\sigma }(u)=|u|-\sqrt{4(Q-1)-\kappa^{2}}+\kappa \arcsin\sqrt{1-\frac{\kappa^{2}}{4(Q-1)}}.$$

In particular, $\tilde{\sigma }(u)$ is a 1-Lipschitz function of *u*, and by definition $\tilde{\sigma }(u)\leq \sigma (u)$. Therefore, by [38], Proposition 1.2 and the fact that $|{\text{m}}_{⊥}^{\varepsilon }|\to 0$ in *L*^1^(*Ω*), proved in Step 1, we have

4.18 $$\begin{array}{rl} \underset{\varepsilon \to 0}{lim inf}E_{\varepsilon }({\text{m}}_{\varepsilon }) & \geq \underset{\varepsilon \to 0}{lim inf}\left(\int_{\Omega }|\nabla u_{\varepsilon }|\;d^{2}r+\int_{\partial \Omega }\tilde{\sigma }({\tilde{u}}_{\varepsilon })\;dH^{1}(r)\right)-2\int_{\Omega }h_{∥}^{0}m_{∥}^{0}\;d^{2}r\\ & \geq \int_{\Omega }|\nabla u_{0}|\;d^{2}r+\int_{\partial \Omega }\tilde{\sigma }({\tilde{u}}_{0})\;dH^{1}(r)-2\int_{\Omega }h_{∥}^{0}m_{∥}^{0}\;d^{2}r, \end{array}$$

where $u_{0}\in BV(\Omega ;\{-2\sqrt{Q-1}+\frac{1}{2}\pi \kappa ,2\sqrt{Q-1}-\frac{1}{2}\pi \kappa \})$ and *u*_*ε*_→*u*_0_ in *L*^1^(*Ω*). In (4.18), the first integral in the last line denotes the total variation of *u*_0_, and the second term is understood as an integral of the trace of a BV function [53]. Note that by (4.17) we have $\tilde{\sigma }({\tilde{u}}_{0})=\sigma_{\mathrm{edge}}$ and $|\nabla u_{0}|=\frac{1}{2}\sigma_{\mathrm{wall}}|\nabla m_{∥}^{0}|$, after straightforward algebra. Therefore, the last inequality is equivalent to

4.19 $$\underset{\varepsilon \to 0}{lim inf}E_{\varepsilon }({\text{m}}_{\varepsilon })\geq \frac{\sigma_{\mathrm{wall}}}{2}\int_{\Omega }|\nabla m_{∥}^{0}|\;d^{2}r+\sigma_{\mathrm{edge}}H^{1}(\partial \Omega )-2\int_{\Omega }h_{∥}^{0}m_{∥}^{0}\;d^{2}r,$$

which coincides with (4.5) [53].

*Step 3*: *Upper bound*. Without loss of generality, we may assume *h*_∥_=0 and **h**_⊥_=0. As we have to preserve the constraint |**m**|=1, we will construct an upper bound, using the angle variables *θ* and *ϕ*. Namely, we define m=(sin⁡θcos⁡ϕ,sin⁡θsin⁡ϕ,cos⁡θ) and rewrite the energy in (4.2) in terms of *θ* and *ϕ* (assumed to be sufficiently smooth) as follows:

4.20 $$\begin{array}{rl} E(\text{m}) & =\int_{\Omega }(\varepsilon |\nabla \theta {|}^{2}+\varepsilon \sin^{2}\theta |\nabla \phi {|}^{2}+\varepsilon^{-1}(Q-1)\sin^{2}\theta )\;d^{2}r\\ & \;+\kappa \int_{\Omega }(\sin\theta \cos\theta -\theta )\nabla \cdot \text{v}(\phi )\;d^{2}r+\kappa \int_{\partial \Omega }\theta \text{v}(\phi )\cdot \nu \;dH^{1}(r), \end{array}$$

where v(ϕ)=(cos⁡ϕ,sin⁡ϕ), and we used integration by parts.

Let *Ω*^±^ be defined as in (4.4) with $m_{∥}=m_{∥}^{0}$. Without loss of generality, we assume that ∂*Ω^+^ has *C*^2^ regularity, and that ∂*Ω^+^ intersects ∂*Ω* transversally, if at all. We define

4.21 $$\theta_{*}(x)=\left\{ \begin{array}{ll} 0 & x\in \Omega^{+}\\ \pi & x\in \Omega^{-} \end{array}\right.\;\mathrm{and}\;\theta_{b}(x)=\left\{ \begin{array}{ll} \theta_{0}^{*} & x\in \partial \Omega \setminus \partial \Omega^{-}\\ \pi -\theta_{0}^{*} & x\in \partial \Omega \setminus \partial \Omega^{+} \end{array}\right.,$$

where $\theta_{0}^{*}$ is defined in (3.32), and take a sequence of $\theta_{\varepsilon }\in C^{1}(\bar{\Omega })$ such that

4.22 $$0\leq \theta_{\varepsilon }\leq \pi ,\;\theta_{\varepsilon }\to \theta_{*}\text{ in }L^{1}(\Omega ),\;\theta_{\varepsilon }\to \theta_{b}\text{ in }L^{1}(\partial \Omega ).$$

Note that we also have *θ*_*ε*_→θ_*_ in *L*^*q*^(*Ω*) for every *q*>1.

Now, for a fixed 1<*p*<2, we take two functions *ϕ*^±^_*_∈*W*^1,*p*^(*Ω*^±^) with values in [0,2*π*) such that

4.23 $$\begin{array}{r} \text{v}({\tilde{\phi }}_{*}^{\pm }(x))=\mp \nu_{\Omega^{\pm }}(x)\;\text{for a.e.}\;x\in \partial \Omega^{\pm }, \end{array}$$

where *ν*_*Ω*^±^_ is the outward normal to *Ω*^±^ and ${\tilde{\phi }}_{*}^{\pm }$ are the traces of *ϕ*^±^_*_ on ∂*Ω*^±^. Such functions exists, for example, by [54], Theorem 2, as ${\tilde{\phi }}_{*}^{\pm }$ are *C*^1^ functions of the arclength, except at a finite number of isolated points where they have jump discontinuities, and, hence, belong to the appropriate Besov spaces in the assumptions of [54]. Next, we define ϕ_*_∈*W*^1,*p*^(*Ω*) as

4.24 $$\begin{array}{r} \phi_{*}(x)=\left\{ \begin{array}{ll} \phi_{*}^{-}(x) & x\in \Omega^{-}\\ \phi_{*}^{+}(x) & x\in \Omega^{+} \end{array}\right. \end{array}$$

and observe that by construction we have

4.25 $$\text{v}({\tilde{\phi }}_{*})=\left\{ \begin{array}{ll} \nu_{\Omega } & \text{on}\;\partial \Omega^{-}\cap \partial \Omega \\ -\nu_{\Omega } & \text{on}\;\partial \Omega^{+}\cap \partial \Omega \\ \nu_{*} & \text{on}\;\partial^{*}\Omega^{+} \end{array}\right.,$$

where *ν*’s are the corresponding outward normals to the respective boundaries and ${\tilde{\phi }}_{*}$ is the trace of ϕ_*_ on those boundaries. We can then construct, using a regularization and a diagonal argument, a sequence of $\phi_{\varepsilon }\in C^{1}(\bar{\Omega })$ such that

4.26 $$\phi_{\varepsilon }\to \phi_{*}\text{ in }W^{1,p}(\Omega )\;\mathrm{and}\;\varepsilon |\nabla \phi_{\varepsilon }{|}^{2}\to 0\text{ in }L^{1}(\Omega ).$$

It is then clear that, as *ε*→0, we have

4.27 $$\begin{array}{rl} & \int_{\Omega }\theta_{\varepsilon }\nabla \cdot \text{v}(\phi_{\varepsilon })\;d^{2}r\to \pi \int_{\Omega^{-}}\nabla \cdot \text{v}(\phi_{*})\;d^{2}r=\pi H^{1}(\partial^{*}\Omega^{+})+\pi H^{1}(\partial \Omega^{-}\cap \partial \Omega ), \end{array}$$

4.28 $$\begin{array}{rl} & \int_{\Omega }\sin\theta_{\varepsilon }\cos\theta_{\varepsilon }\nabla \cdot \text{v}(\phi_{\varepsilon })\;d^{2}r\to 0 \end{array}$$

4.29 $$\begin{array}{rl} \;\mathrm{and}\; & \int_{\partial \Omega }\theta_{\varepsilon }\text{v}(\phi_{\varepsilon })\cdot \nu \;dH^{1}(r)\to -\theta_{0}^{*}H^{1}(\partial \Omega^{+}\cap \partial \Omega )+(\pi -\theta_{0}^{*})H^{1}(\partial \Omega^{-}\cap \partial \Omega ). \end{array}$$

Passing to the limit as *ε*→0 in the energy (4.20) and combining the terms, we obtain

4.30 $$\begin{array}{rl} \underset{\varepsilon \to 0}{lim sup}E({\text{m}}_{\varepsilon }) & =\underset{\varepsilon \to 0}{lim sup}\int_{\Omega }(\varepsilon |\nabla \theta_{\varepsilon }{|}^{2}+\varepsilon^{-1}(Q-1)\sin^{2}\theta_{\varepsilon })\;d^{2}r\\ & \;-\pi \kappa H^{1}(\partial^{*}\Omega^{+})-\kappa \theta_{0}^{*}H^{1}(\partial \Omega ). \end{array}$$

In order to conclude, we need to construct a sequence of $\theta_{\varepsilon }\in C^{1}(\bar{\Omega })$ satisfying (4.22) such that

4.31 $$\underset{\varepsilon \to 0}{lim sup}\int_{\Omega }(\varepsilon |\nabla \theta_{\varepsilon }{|}^{2}+\varepsilon^{-1}(Q-1)\sin^{2}\theta_{\varepsilon })\;d^{2}r=E_{0}(m_{∥})+\pi \kappa H^{1}(\partial^{*}\Omega^{+})+\kappa \theta_{0}^{*}H^{1}(\partial \Omega ).$$

This construction was done in a more general setting in [40], Lemma 2) and, therefore, using this result we conclude that $\underset{\varepsilon \to 0}{lim sup}E({\text{m}}_{\varepsilon })=E_{0}(m_{∥})$, where ${\text{m}}_{\varepsilon }=(\sin\theta_{\varepsilon }\cos\phi_{\varepsilon },\sin\theta_{\varepsilon }\sin\phi_{\varepsilon },\cos\theta_{\varepsilon })$ and (*θ*_*ε*_,*ϕ*_*ε*_) are as above. ▪

As an immediate consequence of *Γ*-convergence, we have the following asymptotic characterization of minimizers of the energy *E*_*ε*_ in terms of the minimizers of *E*_0_.

**Corollary 4.2.**

*Under the assumptions of theorem 4.1, let* $m_{\varepsilon }=(m_{⊥}^{\varepsilon },m_{∥}^{\varepsilon })\in H^{1}(\Omega ;S^{2})$ *be a sequence of minimizers of E*_*ε*_. *Then, after extracting a subsequence, we have* $m_{∥}^{\varepsilon }\to m_{∥}^{0}$ *and* $|m_{⊥}^{\varepsilon }|\to 0$ *in L*^1^(*Ω*), *where* $m_{∥}^{0}\in BV(\Omega ;\{-1,1\})$ *is a minimizer of E*_0_.

For a simple example of an application of the above result, consider the problem on the domain *Ω*=(−2*L*,2*L*)×(−*L*,*L*), corresponding to the geometry in figure 3, in the presence of an applied field $h_{∥}^{0}=-\alpha x$, with *L*>0 and *α*>0. Then it is easy to see that the minimizer of *E*_0_ is *m*_∥_=−sgn(*x*) for all *α* sufficiently large, as in figure 3.

We note that by classical results for problems with prescribed mean curvature (e.g. [55] and references therein), the minimizers of *E*_0_ are functions, whose jump set $\Gamma \subset \bar{\Omega }$ is a union of finitely many *C*^1,1^ curve segments satisfying weakly the equation

4.32 $$\sigma_{\mathrm{wall}}K(x)=4h_{∥}^{0}(x),\;x\in \Gamma \cap \Omega ,\;\Gamma^{\prime }(x)⊥\partial \Omega ,\;x\in \Gamma \cap \partial \Omega ,$$

where *K* is the curvature of *Γ*, positive if the set *Ω*^+^ is convex, and the prime denotes arclength derivative. Physically, these are interpreted as the Dzyaloshinskii domain walls separating the domains of opposite out-of-plane magnetization under the external applied field. We also note that the limit energy *E*_0_ contains a contribution from the edge domain walls, which, however, is independent of the magnetization orientation near the edge and thus only adds a constant term to the energy.

**Remark 4.3**

We note that by the results of [39], we can also say that if $m_{∥}^{0}$ is an isolated local minimizer of *E*_0_, then there exists a sequence of local minimizers **m**_*ε*_ of *E*_*ε*_ such that $m_{∥}^{\varepsilon }\to m_{∥}^{0}$ and ${\text{m}}_{⊥}^{\varepsilon }\to 0$ in *L*^1^(*Ω*).

Before concluding this section, let us comment on some topological issues related to the result in theorem 4.1. We note that our upper construction in theorem 4.1 uses the magnetization configurations that have topological degree zero. This has to do with the representation of the test configurations **m**_*ε*_ adopted in the proof in terms of the angle variables (*θ*_*ε*_,*ϕ*_*ε*_), which are assumed to be of class *C*^1^ up to the boundary. Therefore, the proof does not immediately extend to the admissible classes with prescribed topological degree distinct from zero. This is not a problem, however, in view of the fact that away from the domain walls one could insert skyrmion profiles [33], suitably localized, into our test functions to prescribe a fixed topological degree for *ε* sufficiently small. Our result would then not be altered, in view of the fact that in the considered scaling the energy of a skyrmion is a lower order perturbation to that of chiral walls. In other words, under the considered scaling assumptions our energy does not see magnetic skyrmions.

## 5. Discussion

To summarize, we have analysed the basic domain wall profiles in the local version of the micromagnetic modelling framework containing DMI, which is governed by the energy in (2.2). Specifically, we performed an analysis of the one-dimensional energy minimizing configurations on the whole line and on half-line and showed that the magnetization profiles expected from the physical considerations based on specific *ansätze* are indeed the unique global energy minimizers for $|\kappa |<\sqrt{Q-1}$. This is slightly below (approx. 30%) the threshold value of $|\kappa |=\kappa_{c}=(4/\pi )\sqrt{Q-1}$, beyond which helical structures emerge. Our methods rely on a sharp Modica–Mortola type inequality and do not extend to the narrow range of $\sqrt{Q-1}\leq |\kappa |<(4/\pi )\sqrt{Q-1}$. It is natural to expect that our result persists all the way to |*κ*|=*κ*_c_, but to justify this statement one would need to develop new analysis tools for the vectorial variational problem associated with the domain walls.

Our one-dimensional analysis in §3 identified two basic types of chiral domain walls: the interior and the edge domain walls. These one-dimensional domain wall solutions are the building blocks of the more complicated two-dimensional magnetization configurations in ultrathin films subjected to sufficiently small applied magnetic fields. This can be seen from the analysis of *Γ*-convergence of the energy in (4.2) performed in §4. Either global or local energy minimizers for *ε*≪1 may then be approximated by those of the energy in (4.3), which determines the geometry of the magnetic domains in the sample. Our findings indicate that in the considered limit the magnetization configurations solve the prescribed mean curvature problem in (4.32), again, for $|\kappa |<\sqrt{Q-1}$. We note that our variational setting could similarly be used to study the gradient flow dynamics governed by (4.2) (for a related study, see [40]). Other physical effects, however, need to be incorporated to account for some unusual properties of chiral domain walls such as their tilt in sufficiently strong external fields [22,35].

Finally, we would like to comment on the assumptions that lead to the model in (4.2), and on its possible generalizations. As was already mentioned, this energy functional is local, with the effect of the stray field surviving in the renormalized magnetocrystalline anisotropy term only. This is justified in the limit of arbitrarily thin ferromagnetic films [45]. In practice, this contribution is only the leading order term in the expansion of the energy in the film thickness for films whose thickness is less than the exchange length ℓ_ex_ of the material. Going to the next order, two types of contributions appear. The first is the one coming from the sample boundary. In the limit of the dimensionless film thickness *δ*=*d*/ℓ_ex_ going to zero, this contribution becomes local and adds an extra penalty term for the in-plane component of the magnetization at the edge [56]:

5.1 $$E_{\varepsilon }^{\mathrm{edge}}(\text{m})=\frac{\delta |\ln\delta |}{2\pi }\int_{\partial \Omega }{\left(\nu \cdot {\text{m}}_{⊥}\right)}^{2}\;dH^{1}(r),$$

where *ν* is the outward unit normal to ∂*Ω*. Here we took into account that in a perpendicular material the magnetic ‘charge’ at the sample boundary would be smeared on the scale of ℓ_ex_. In the interior, the leading order contribution from the stray field energy beyond the shape anisotropy can be shown to be [46]

5.2 $$\begin{array}{rl} E_{\varepsilon }^{\mathrm{bulk}}(\text{m}) & =-\frac{\delta }{8\pi }\int_{\Omega }\int_{\Omega }\frac{{\left(m_{∥}(\text{r})-m_{∥}({\text{r}}^{\prime })\right)}^{2}}{|\text{r}-{\text{r}}^{\prime }{|}^{3}}\;d^{2}r\;d^{2}r^{\prime }\\ & \;+\frac{\delta }{4\pi }\int_{\Omega }\int_{\Omega }\frac{\nabla \cdot {\text{m}}_{⊥}(\text{r})\;\nabla \cdot {\text{m}}_{⊥}({\text{r}}^{\prime })}{|\text{r}-{\text{r}}^{\prime }|}\;d^{2}r\;d^{2}r^{\prime }. \end{array}$$

Furthermore, for $\delta =\lambda |\ln\varepsilon {|}^{-1}$ it was shown in the case *κ*=0 and periodic boundary conditions in the plane that as *ε*→0 the effect of the stray field energy is to renormalize the one-dimensional wall energy to a lower value, as long as $\lambda <\lambda_{c}=2\pi \sqrt{Q-1}$ [46]. It is natural to expect from the results of [46] that, as *ε*→0, the wall energy for *κ*>0 will become

5.3 $$\sigma_{\mathrm{wall}}=4\sqrt{Q-1}-\pi \kappa -\frac{2\lambda }{\pi }.$$

Similarly, one would expect that in this regime the edge wall energy *σ*_edge_ would also be renormalized to minimize the sum of the exchange, anisotropy, DMI energies (all contained in (4.2)) and the stray field energy contributions from (5.1) and (5.2). This study is currently underway. At the same time, for *λ*>*λ*_c_ one expects spontaneous onset of milti-domain magnetization patterns and qualitatively new system behaviour (for a recent experimental illustration, see [15]).

## References

[1] Prinz GA 1998 Magnetoelectronics. *Science* 282, 1660–1663. (doi:10.1126/science.282.5394.1660) 9831549 10.1126/science.282.5394.1660

[2] Zutic I, Fabian J, Das Sarma S 2004 Spintronics: fundamentals and applications. *Rev. Mod. Phys.* 76, 323–410. (doi:10.1103/RevModPhys.76.323)

[3] Allwood DA, Xiong G, Faulkner CC, Atkinson D, Petit D, Cowburn RP 2005 Magnetic domain-wall logic. *Science* 309, 1688–1692. (doi:10.1126/science.1108813) 16151002 10.1126/science.1108813

[4] Bader SD, Parkin SSP 2010 Spintronics. *Ann. Rev. Condens. Matter Phys.* 1, 71–88. (doi:10.1146/annurev-conmatphys-070909-104123)

[5] Brataas A, Kent AD, Ohno H 2012 Current-induced torques in magnetic materials. *Nat. Mater.* 11, 372–381. (doi:10.1038/nmat3311) 22522637 10.1038/nmat3311

[6] Fert A, Cros V, Sampaio J 2013 Skyrmions on the track. *Nat. Nanotechnol.* 8, 152–156. (doi:10.1038/nnano.2013.29) 23459548 10.1038/nnano.2013.29

[7] Nagaosa N, Tokura Y 2013 Topological properties and dynamics of magnetic skyrmions. *Nat. Nanotechnol.* 8, 899–911. (doi:10.1038/nnano.2013.243) 24302027 10.1038/nnano.2013.243

[8] Chen G, Ma T, N’Diaye AT, Kwon H, Won C, Wu Y, Schmid AK 2013 Tailoring the chirality of magnetic domain walls by interface engineering. *Nat. Commun.* 4, 1–6. (doi:10.1038/ncomms3671) 10.1038/ncomms3671 24154595

[9] von Bergmann K, Kubetzka A, Pietzsch O, Wiesendanger R 2014 Interface-induced chiral domain walls, spin spirals and skyrmions revealed by spin-polarized scanning tunneling microscopy. *J. Phys. Condens. Matter* 26, 394002 (doi:10.1088/0953-8984/26/39/394002) 25214495 10.1088/0953-8984/26/39/394002

[10] Matsukura F, Tokura Y, Ohno H 2015 Control of magnetism by electric fields. *Nat. Nanotechnol.* 10, 209–220. (doi:10.1038/nnano.2015.22) 25740132 10.1038/nnano.2015.22

[11] Parkin SSP, Hayashi M, Thomas L 2008 Magnetic domain-wall racetrack memory. *Science* 320, 190–194. (doi:10.1126/science.1145799) 18403702 10.1126/science.1145799

[12] Bogdanov AN, Yablonskii DA 1989 Thermodynamically stable ‘vortices’ in magnetically ordered crystals. The mixed state of magnets. *Sov. Phys. JETP* 68, 101–103.

[13] Bogdanov A, Hubert A 1994 Thermodynamically stable magnetic vortex states in magnetic crystals. *J. Magn. Magn. Mater.* 138, 255–269. (doi:10.1016/0304-8853(94)90046-9)

[14] Heinze S, von Bergmann K, Menzel M, Brede J, Kubetzka A, Wiesendanger R, Bihlmayer G, Blugel S 2011 Spontaneous atomic-scale magnetic skyrmion lattice in two dimensions. *Nat. Phys.* 7, 713–718. (doi:10.1038/nphys2045)

[15] Woo S *et al.* 2016 Observation of room-temperature magnetic skyrmions and their current-driven dynamics in ultrathin metallic ferromagnets. *Nat. Mat.* 15, 501–506. (doi:10.1038/nmat4593) 10.1038/nmat4593 26928640

[16] Zhang X, Ezawa M, Zhou Y 2015 Magnetic skyrmion logic gates: conversion, duplication and merging of skyrmions. *Sci. Rep.* 5, 9400 (doi:10.1038/srep09400) 25802991 10.1038/srep09400 PMC4371840

[17] Braun H-B 2012 Topological effects in nanomagnetism: from superparamagnetism to chiral quantum solitons. *Adv. Phys.* 61, 1–116. (doi:10.1080/00018732.2012.663070)

[18] Thiaville A, Rohart S, Jué E, Cros V, Fert A 2012 Dynamics of Dzyaloshinskii domain walls in ultrathin magnetic films. *Europhys. Lett.* 100, 57002 (doi:10.1209/0295-5075/100/57002)

[19] Rohart S, Thiaville A 2013 Skyrmion confinement in ultrathin film nanostructures in the presence of Dzyaloshinskii–Moriya interaction. *Phys. Rev. B* 88, 184422 (doi:10.1103/PhysRevB.88.184422)

[20] Goussev A, Lund RG, Robbins JM, Slastikov V, Sonnenberg C 2013 Domain wall motion in magnetic nanowires: an asymptotic approach. *Proc. R. Soc. A* 469, 20130308 (doi:10.1098/rspa.2013.0308) 24353468 10.1098/rspa.2013.0308 PMC3857867

[21] Sampaio J, Cros V, Rohart S, Thiaville A, Fert A 2013 Nucleation, stability and current-induced motion of isolated magnetic skyrmions in nanostructures. *Nat. Nanotechnol.* 8, 839–844. (doi:10.1038/nnano.2013.210) 24162000 10.1038/nnano.2013.210

[22] Boulle O, Rohart S, Buda-Prejbeanu LD, Jué E, Miron IM, Pizzini S, Vogel J, Gaudin G, Thiaville A 2013 Domain wall tilting in the presence of the Dzyaloshinskii–Moriya interaction in out-of-plane magnetized magnetic nanotracks. *Phys. Rev. Lett.* 111, 217203 (doi:10.1103/PhysRevLett.111.217203) 24313522 10.1103/PhysRevLett.111.217203

[23] Stepanova M, Dew S (eds). 2012 *Nanofabrication: techniques and principles*. Vienna, Austria: Springer.

[24] Heinrich B, Cochran JF 1993 Ultrathin metallic magnetic films: magnetic anisotropies and exchange interactions. *Adv. Phys.* 42, 523–639. (doi:10.1080/00018739300101524)

[25] Ikeda S *et al.* 2010 A perpendicular-anisotropy CoFeB–MgO magnetic tunnel junction. *Nat. Mater.* 9, 721–724. (doi:10.1038/nmat2804) 20622862 10.1038/nmat2804

[26] Dzyaloshinskii I 1958 A thermodynamic theory of ‘weak’ ferromagnetism of antiferromagnetics. *J. Phys. Chem. Solids* 4, 241–255. (doi:10.1016/0022-3697(58)90076-3)

[27] Moriya T 1960 Anisotropic superexchange interaction and weak ferromagnetism. *Phys. Rev.* 120, 91–98. (doi:10.1103/PhysRev.120.91)

[28] Fert A 1990 Magnetic and transport-properties of metallic multilayers. *Mater. Sci. Forum* 59, 439–480. (doi:10.4028/www.scientific.net/MSF.59-60.439)

[29] Hrabec A, Porter NA, Wells A, Benitez MJ, Burnell G, McVitie S, McGrouther D, Moore TA, Marrows CH 2014 Measuring and tailoring the Dzyaloshinskii–Moriya interaction in perpendicularly magnetized thin films. *Phys. Rev. B* 90, 020402 (doi:10.1103/PhysRevB.90.020402)

[30] Bode M *et al.* 2007 Chiral magnetic order at surfaces driven by inversion asymmetry. *Nature* 447, 190–193. (doi:10.1038/nature05802) 17495922 10.1038/nature05802

[31] Romming N, Hanneken C, Menzel M, Bickel JE, Wolter B, von Bergmann K, Kubetzka A, Wiesendanger R 2013 Writing and deleting single magnetic skyrmions. *Science* 341, 636–639. (doi:10.1126/science.1240573) 23929977 10.1126/science.1240573

[32] Emori S, Bauer U, Ahn S-M, Martinez E, Beach GSD 2013 Current-driven dynamics of chiral ferromagnetic domain walls. *Nat. Mat.* 12, 611–616. (doi:10.1038/nmat3675) 10.1038/nmat3675 23770726

[33] Melcher C 2014 Chiral skyrmions in the plane. *Proc. R. Soc. A* 470, 1–17. (doi:10.1098/rspa.2014.0394)

[34] Hubert A, Schäfer R 1998 *Magnetic domains*. Berlin, Germany: Springer.

[35] Muratov CB, Slastikov VV, Tretiakov OA In preparation. Theory of tilted Dzyaloshinskii walls in the presence of in-plane magnetic fields.

[36] DeSimone A, Kohn RV, Müller S, Otto F 1998 Recent analytical developments in micromagnetics. In *The science of hysteresis, volume 2 of physical modelling, micromagnetics, and magnetization dynamics* (eds G Bertotti, ID Mayergoyz), pp. 269–381. Oxord, UK: Academic Press.

[37] Modica L 1987 The gradient theory of phase transitions and the minimal interface criterion. *Arch. Rational Mech. Anal.* 98, 123–142. (doi:10.1007/BF00251230)

[38] Modica L 1987 Gradient theory of phase transitions with boundary contact energy. *Ann. Inst. Henri Poincaré. Anal. Non Linéaire* 4, 487–512.

[39] Kohn RV, Sternberg P 1989 Local minimisers and singular perturbations. *Proc. R. Soc. Edinb. A* 111, 69–84. (doi:10.1017/S0308210500025026)

[40] Owen NC, Rubinstein J, Sternberg P 1990 Minimizers and gradient flows for singularly perturbed bi-stable potentials with a Dirichlet condition. *Proc. R. Soc. Lond. A* 429, 505–532. (doi:10.1098/rspa.1990.0071)

[41] Fonseca I, Tartar L 1989 The gradient theory of phase transitions for systems with two potential wells. *Proc. R. Soc. Edinb. A* 111, 89–102. (doi:10.1017/S030821050002504X)

[42] Sternberg P 1991 Vector-valued local minimizers of nonconvex variational problems. *Rocky Mt. J. Math.* 21, 799–807. (doi:10.1216/rmjm/1181072968)

[43] Fert A, Levy PM 1980 Role of anisotropic exchange interactions in determining the properties of spin-glasses. *Phys. Rev. Lett.* 44, 1538–1541. (doi:10.1103/PhysRevLett.44.1538)

[44] Crépieux A, Lacroix C 1998 Dzyaloshinsky–Moriya interactions induced by symmetry breaking at a surface. *J. Magn. Magn. Mater.* 182, 341–349. (doi:10.1016/S0304-8853(97)01044-5)

[45] Gioia G, James RD 1997 Micromagnetics of very thin films. *Proc. R. Soc. Lond. A* 453, 213–223. (doi:10.1098/rspa.1997.0013)

[46] Knüpfer H, Muratov CB, Nolte F In preparation. Magnetic domains in thin ferromagnetic films with strong perpendicular anisotropy.

[47] Chermisi M, Muratov CB 2013 One-dimensional Néel walls under applied external fields. *Nonlinearity* 26, 2935–2950. (doi:10.1088/0951-7715/26/11/2935)

[48] Brezis H 2011 *Functional analysis, Sobolev spaces and partial differential equations*. Berlin, Germany: Springer.

[49] Kohn RV 2007 Energy-driven pattern formation. In *Int. congress of mathematicians*, vol. I, pp. 359–383. Zürich, Switzerland: EMS.

[50] Wilson MN, Karhu EA, Lake DP, Quigley AS, Meynell S, Bogdanov AN, Fritzsche H, Rößler UK, Monchesky TL 2013 Discrete helicoidal states in chiral magnetic thin films. *Phys. Rev. B* 88, 214420 (doi:10.1103/PhysRevB.88.214420)

[51] Meynell SA, Wilson MN, Fritzsche H, Bogdanov AN, Monchesky TL 2014 Surface twist instabilities and skyrmion states in chiral ferromagnets. *Phys. Rev. B* 90, 014406. (doi:10.1103/PhysRevB.90.014406)

[52] Lieb EH, Loss M 2010 *Analysis*. Providence, RI: American Mathematical Society.

[53] Ambrosio L, Fusco N, Pallara D 2000 *Functions of bounded variation and free discontinuity problems*. Oxford Mathematical Monographs New York, NY: The Clarendon Press.

[54] Marschall J 1987 The trace of Sobolev-Slobodeckij spaces on Lipschitz domains. *Manuscr. Math.* 58, 47–65. (doi:10.1007/BF01169082)

[55] Maggi F 2012 *Sets of finite perimeter and geometric variational problems*. Cambridge Studies in Advanced Mathematics 135 Cambridge, UK: Cambridge University Press.

[56] Kohn RV, Slastikov VV 2005 Another thin-film limit of micromagnetics. *Arch. Ration. Mech. Anal.* 178, 227–245. (doi:10.1007/s00205-005-0372-7)

